# Measuring and Analyzing Waiting Time Indicators of Patients’ Admitted in Emergency Department: A Case Study

**DOI:** 10.5539/gjhs.v8n1p143

**Published:** 2015-05-15

**Authors:** Saeed Amina, Ahmad Barrati, Jamil Sadeghifar, Marzyeh Sharifi, Zahra Toulideh, Hasan Abolghasem Gorji, Negar Feazbakhsh

**Affiliations:** 1 School of Health management and Information Sciences, Iran University of Medical Sciences, Tehran, Iran; 2 Department of Health Education, School of Health, Ilam University of Medical Sciences, Ilam, Iran; 3 Department of Health Management, School of Health Management and Information Sciences, International Campus (IUMS-IC), Iran University of Medical Sciences, Tehran, Iran; 4 Health Economics and Management Research Center, Iran University of Medical Sciences, Tehran, Iran; 5 Faculty of Nursing & Midwifery, Urmia University of Medical Sciences, Urmia, Iran & Faculty of Nursing- Midwifery, Kurdistan University of Medical Sciences, Sanandaj, Iran

**Keywords:** waiting time, indicators, workflow, emergency department, hospital

## Abstract

**Background & Aims:**

Measuring and analyzing of provided services times in Emergency Department is the way to improves quality of hospital services. The present study was conducted with aim measuring and analyzing patients waiting time indicators in Emergency Department in a general hospital in Iran.

**Material & Methods:**

This cross-sectional, observational study was conducted during April to September 2012. The study population consisted of 72 patients admitted to the Emergency Department at Baharlo hospital. Data collection was carried out by workflow forms. Data were analyzed by t. test and ANOVA.

**Results:**

The average waiting time for patients from admission to enter the triage 5 minutes, the average time from triage to physician visit 6 minute and the average time between examinations to leave ED was estimated 180 minutes. The total waiting time in the emergency department was estimated at about 210 minutes. The significant correlation between marital status of patients (P=0.03), way of arrive to ED (P=0.02) and type of shift work (P=0.01) with studied time indicators were observed.

**Conclusion:**

According to results and comparing with similar studies, the average waiting time of patients admitted to the studied hospital is appropriate. Factors such as: Utilizing clinical governance system and attendance of resident Emergency Medicine Specialist have performed an important role in reducing of waiting times in ED.

## 1. Introduction

Crowd in the Emergency Department is considered as a challenging issue worldwide (Akcali, Coˆté & Lin, 2006; Xie, Chaussalet, & Millard, 2006) and this issue deranges the capability of hospitals to present the emergency attention in a logical time format (Rowe et al. 2006). Guttmann, Schull, Vermeulen, & Stukel, (2011) after studying more than 20 million visited patients in the emergency departments of Ontario for 5 years, said that mortality risk and hospital re-admission will increase according to crowd level in the emergency department. They also estimated if the average waiting time for emergency services reduce to an hour, it potentially prevents the death of 150 patients (Guttmann, 2011). Therefore; the speed of delivering emergency services in hospitals has a noticeable consequence in reduction of disability and mortality rate. Any minutes are vital to affect the interval between death and severe disabilities and or a fruitful life (Heydaranlou, Khaghani-Zadeh, Ebadi, Sirati-Nir, & Aghdasi Mehr-Abad, 2008).

Some recent studies have shown that crowdedness in emergency department depend on many important factors in which patients’ not leaving the ward due to reasons (Bagust, Place, & Posnett 1999; Han J. H. et al. 2007). Long waiting time in Emergency Department may cause undesirable effects in the practicality of clinical services and it also lead to dissatisfaction of patients and their attendants (Gol Aghayi, Sarmadian, Rafii, & Nejat, 2008). Many researches shown that the patients’ waiting time to reach appropriate services is one of the most important criteria of evaluating the Emergency Departments in hospitals (Don, Danny, & Kennedy, 2003; Yoon, Steiner, & Reinhardt, 2003; Viccellio et al., 2008; Casalino et al., 2012; Nordstrom et al., 2012; Hashemi et al., 2013). Among the most imperative standard criteria to evaluate the quality of services in the Emergency Department, include some crucial times such as: time intervals between patients’ admission to triage, from triage to physician’s visit, physician’s visit to examination and medical treatment and at the end to leave ED which all of these criteria need to be considered through the time measuring indicator (Salluzzo, Mayer, Strauss, & Kidd, 1997; Hashemi et al., 2013).

So far, many researches have been conducted to measure the waiting time for emergency patients in association between patients’ satisfaction and the quality of both medical and health care consideration. Hosseini, Shaker, Ghafouri and Shokraneh (2010) reported a standard average time between triage and the treatment phase in a study that they accomplished in one of Tehran hospital site, but stile few data are available on ED waiting times among Iranian public hospitals serving cares for patients. In a research was conducted by Movahed-Nia, et al. (2013), they declared that the presence of emergency resident doctor and the committee of patients’ illness determination were both the appropriate key factors in the waiting time in the Emergency Department in Tehran Firoozgar Hospital. University of California has done a research, for two years, in 30 California EDs Emergency Departments to consider the patients’ waiting time and adverse to a result: patients have waited for an average 56 minutes, visited by the physician (Lambe et al., 2003).

Current study is conducted with the aim of measuring and analyzing the workflow of the patients in the Emergency Department in Baharlo hospital and also determining the waiting time indicators between triage and physician’s visit, physician’s visit and examinations and examinations to leave ED. In addition, one more purpose of this study is to consider the connection between time indicators and some patients’ characteristics, severity of patients’ condition, working shift, attendance and the ways of arrival to the Emergency Department. As informed by these time indicators, the present condition and the average time that patients spend in the emergency sections, it’s necessary to design and plan some suitable and powerful interventions in order to improve both clinical practicality and patients’ satisfaction.

## 2. Materials and Methods

This cross-sectional, observational study was conducted during April to September 2012. Numbers of 72 patients admitted to the Emergency Department at Baharlo hospital, affiliated to Tehran University of Medical Science were selected randomly to be studied. In order to consider the effects of different factors on the workflow of the patients, we tried to choose patients from different shifts and different week days. On this basis, half of the patients were chosen from morning shifts and the other half was from the afternoon and night shifts.

Data gathering was fulfilled by the workflow forms which was consist of two parts. First part includes: the demographic characteristics of the patients such as gender, age, marital status, education, and severity of the illness, attending visit time, reasons of leaving the emergency department and also the ways of arrival to the emergency department. Second part includes: 1). Waiting time for patients from admission to enter the triage, 2). The average time from triage to physician visit, 3). The average time between examinations to leave ED, 4). The total waiting time in the emergency department. Data about the demographic characteristics was gathered by questioning the patients or their attendants. If patients are unwilling to answer the questions, these data was gathered from the nurses. Furthermore, about the second part of the form measuring data was recorded by observing patients’ workflow or using stopwatch. In this study the satisfaction of patients, participants, was either asked from them and in case the patients weren’t in a proper condition, their attendants were asked orally. Data were analyzed using SPSS (Version 18) software followed by t. test and ANOVA. The ethical permission in performing the study issued by the ethical committee and authorities were informed beforehand and made them sure that the results were presented to the hospital while keeping confidentiality of the information.

## 3. Results

Out of the total number of the participants in this study, 37(51.34%) female, 42(58.34%) married, major age group between: 40-60(30.55%) and almost half of them were high school dropout (48.61%). In sum, most of the patients (44.45%) referred to the wards with their attendants and the least percentage (12%) was transferred to hospitals with private ambulance. Also, most of the emergency patients (55.55%) were referred to other wards after first emergency medical considerations. (Table 1)

**Table 1:** Distribution of the admitted patient in the study based on demographic characteristic variation number percentage

Demographic characteristic	Variation	Frequency	Valid percent
Gender	Male	35	48.61
	Female	37	51.39
Marital status	Single	30	41.66
	Married	42	58.34
Education background	Illiterate	19	26.38
	Diploma dropout	35	48.61
	Diploma & higher	18	25.00
Age of the patient	Under 20 years	15	20.83
	20-40	21	29.16
	40-60	22	30.55
	Over 60	14	19.44
Severity of patient condition	Semi-urgent	18	25.00
	Non-urgent	42	58.33
	Urgent	12	16.66
Patient’s reason to leave ED	Discharged	23	31.94
	Transport to the ward	40	55.55
	Transport to other place	5	6.94
	Death	4	5.55
Way of patient’s arrival to the emergency	By an attendant	32	44.45
	In person	20	27.77
	Private ambulance	9	12.50
	Emergency department ambulance	11	15.27
Attendance shift	Morning shift	35	50.00
	Afternoon/night shift	35	50.00

Concerning the waiting time for patients from admission to triage in emergency departments, majority of them (40.2%) between 3-6 minutes and the least of them (2.8%) were triaged less than a minute from the admission. Regarding the time from triage to physician visit in the emergency department, most of them (49.8%) were between 1-6 minutes and the least percentage (1.4%) examined by the specialist, approximately 21 minutes waiting time from the triage. About the time between the examinations to ED, most of them (54.41%) between 3-5 hours and the least percentage (1.4%) were left the ward in less than an hour from the time they visited by the physician. Generally, total waiting time in the emergency department, from the admission to leaving the ED, the most percentage (29.2%) was between 3-4 hours and the least percentage (7%) has left the triage in more than 6 hours (Table 2).

**Table 2:** Statistic correlation between demographic variation and waiting time indicators

No.	Variation	Attendance to triage (P-value)	Triage to examination (P-value)	Examination to leave ED (P-value)	Total time in ward (P-value)
**1**	Gender	0.26	0.77	0.22	0.21
**2**	Marital status	0.81	0.67	0.03	0.03
**3**	Age	0.39	0.07	0.43	0.46
**4**	Education	0.36	0.81	0.98	0.99
**5**	Severity of patient’s condition	0.35	0.31	0.19	0.17
**6**	Reason to leave ED	0.12	0.86	0.44	0.38
**7**	Attendance shift	0.42	0.57	0.01	0.01
**8**	Way of arriving to the emergency	0.39	0.02	0.96	0.97

On the whole, the average waiting time in this hospital from admission to the triage of the patients 5 minutes, triage to the physician examination 6 minutes, examination to leave the ED 180 minutes and the total waiting time in the emergency department was 210 minutes.

The results have revealed that there is a significant difference between patients’ marital status with the average waiting time they spend to receive physician examination and leaving the ED (P=0.03) and also the total waiting time in the emergency department (p=0.01). Therefore, as found by this research, there is another significant difference between the patients waiting time from triage to physician examination and way of arriving to the ED (p=0.02). The results showed significant association of interval between attendance shift and the average total waiting time from examination to leave ED (P<0.001). There was no significant difference between the variable of the age, sex, education background and the way of arriving to the ED with the total waiting time criteria as well (P<0.05), (Table 2).

## 4. Discussion

In the present study, in order to provide the evidence for the management of the Emergency Department of Baharlo hospital, researchers focused to measure the patients’ average waiting time on the basis of the process to receive emergency services. The results of this study can be used to develop and plan appropriate interventions to reduce the patients waiting time in emergency department and as identified indicators in this study. Such a consideration would optimize the effectiveness, applicability and adoption of appropriate indicators by hospitals to maximize the patients and attendants’ satisfaction, reduces stress for the allied profession effort and the effectiveness of clinical, medical treatments and overcrowding as well.

The finding of the present study showed that establishment of emergency medicine results in a more efficacious triage, the average waiting time from admission to triage 5 minutes, from triage to physician examination 6 minutes, examination to leave the ED 180 Minutes and the average total waiting time in the emergency department is estimated 210 minutes. A qualitative study, which is conducted by Tabibi and colleagues (2009) in selected Universities of Medical Sciences in Iran, the average total waiting time of the patients in the emergency department is estimated to be 284 minutes. According to a research conducted by Movahed-Nia and colleagues, in Firoozgar Hospital the average accessible time to achieve a clinical visit in the emergency department is identified 3 minutes and the average waiting time in the emergency to triage is estimated as 305 minutes (Movahednia, Partovishayan & Bastani-tehrani, 2013).

Results by a study was identified the average waiting time in the emergency is reported to be 353 minutes (Jabbari, Jafarian, Khorasani, Ghaffari, & Majlesi, 2011). Also based on some existed references the average standard time the patients stayed in the emergency is noted as 4 hours (Canadian Institute for Health Information, 2005).

Comparing the result of this study with these cited researches has shown that the timing and emergency services in Baharlo Hospital is appropriate, although there are still plenty steps to achieve the ideal criteria, the effective triage section (ward) and the proximity of the Para-clinic units (Radiology, Lab and Pharmacies) to the emergency department requires to reduce the average waiting time to some extent.

The gathered results considering the correlation between the patient’s marital status and their waiting time has shown that there is a significant difference between the waiting time to receive examinations to leave ED and also between the total waiting time in the emergency and the patient’s marital status. It is because married patients ask one or more of their family members to do the paperwork, preparing the medical file, asking for the lab results or going to radiography and on the whole it makes the differences.

The results indicated that there is another significant difference between the patients waiting time from triage to examination and their arrival with emergency ambulances and private ambulances. Patients transported to the emergency with ambulance have spent less time to receive examinations. Perhaps it is because the patients, who are transported with the ambulance to the emergency, make the staff presume it might be a more serious case, so they start the examinations faster since all the actions need to be carried out only by the medical staff/clinicians. As a consequence, the longer waiting time allocates for those patients who attend the emergency in-person and without any attendants. The result of the presented study with the research conducted by Jabbari and colleagues in Alzahra Hospital in Iran show no conformity (Jabbari, Jafarian, Khorasani, Ghaffari, & Majlesi, 2011). In that study, private ambulance was not considered as one of the ways that patients attend the emergency department and it may be the reason for the difference.

Results have also shown that there is a noticeable significant difference between the patients waiting time from physician examination to leaving the ED and the total waiting time in different shifts. According to the number of the people in the emergency who most of them were in the afternoon and night shifts and also the number of the night shift doctors and nurses were less than the morning shift, this difference may cause by the crowdies of the emergency department which is packed full by the patients in the afternoon and night shift. Another factor may affect overcrowding is disability of the staff to respond properly and also the fatigue medical staff in this working shift (Evening/Night). On the other hand, at the weekends because the doctors’ offices are closed, as a result the number of clients will increase. Managerial and administrative system is not entirely functional and responsive; therefore, the speed of paperwork formalities reduces and this may leads to longer waiting time. And in this case study the result is different with Jabbari’s and colleagues’ research (Jabbari, Jafarian, Khorasani, Ghaffari, & Majlesi, 2011).

The severity of clinical condition of the patients in the emergency department of Baharlo Hospital showed that nearly 58% of the consumers of the emergency department include patients with minor diagnoses who were in non-emergency condition in triage platform. If there was way to establish more night clinics, overcrowd situation could be reduced in the ED and providing proper services will be. Moreover, adequacy of staff, clear working relationships between colleagues should be improved in order to attain a reduced crowdies rate in emergency departments.

## 5. Conclusion

According to the results of present study, the average waiting time of the patients to get services and to be triaged seems relatively appropriate but there is still a vast space to fulfill the problems in this field. Factors like recklessness to transport patients to emergency departments, inappropriate primary restoring actions in the accident scene, deficit of educated staff in the medical emergencies in emergency departments, doctors delay to visit the patient, intensive working shifts, inadequate supervision to monitor the residents and interns’ presence in their shifts, the unsuitable internal relationship between emergency and I.C.U to admit the patients with serious medical cases should all be considered.

Some of the limitations of this study are that the sample is not as wide as it should be due to the difficulty and it’s time-taking nature of the study case. It is suggested that in addition to studding the high-data researches in different emergency departments, doing periodic time-measuring studies and considering the effective factors on the services in emergency parts from the different beneficiary’s point of view to focus on the important process of this section, design and performing proper interventions, make a way to reduce the patients waiting time and soon after, provide a way to increase the effectiveness of clinical consideration and patients’ satisfaction as well.
